# A bibliometric analysis and visualization of normal pressure hydrocephalus

**DOI:** 10.3389/fneur.2024.1442493

**Published:** 2024-07-31

**Authors:** Tengwu Chang, Xiaoyuan Huang, Xu Zhang, JinYong Li, Wenju Bai, Jichao Wang

**Affiliations:** ^1^Department of Neurosurgery, People’s Hospital of Xinjiang Uygur Autonomous Region, Urumchi, China; ^2^Xinjiang Second Medical College, Karamay, China

**Keywords:** normal pressure hydrocephalus, CiteSpace, VOSviewer, visual analysis, bibliometric

## Abstract

**Background:**

Normal pressure hydrocephalus (NPH) has drawn an increasing amount of attention over the last 20 years. At present, there is a shortage of intuitive analysis on the trends in development, key contributors, and research hotspots topics in the NPH field. This study aims to analyze the evolution of NPH research, evaluate publications both qualitatively and quantitatively, and summarize the current research hotspots.

**Methods:**

A bibliometric analysis was conducted on data retrieved from the Web of Science Core Collection (WoSCC) database between 2003 and 2023. Quantitative assessments were conducted using bibliometric analysis tools such as VOSviewer and CiteSpace software.

**Results:**

A total of 2,248 articles published between 2003 and 2023 were retrieved. During this period, the number of publications steadily increased. The United States was the largest contributor. The University of Gothenburg led among institutions conducting relevant research. Eide P. K. was the most prolific author. The Journal of Neurosurgery is the leading journal on NPH. According to the analysis of the co-occurrence of keywords and co-cited references, the primary research directions identified were pathophysiology, precise diagnosis, and individualized treatment. Recent research hotspots have mainly focused on epidemiology, the glymphatic system, and CSF biomarkers.

**Conclusion:**

The comprehensive bibliometric analysis of NPH offers insights into the main research directions, highlights key countries, contributors, and journals, and identifies significant research hotspots. This information serves as a valuable reference for scholars to further study NPH.

## Introduction

Normal pressure hydrocephalus (NPH) is a significant condition that impacts the physical and mental health of the elderly, first reported by Adams et al. (1) in 1965. It is characterized by normal cerebrospinal fluid (CSF) pressure, ventricular enlargement, and a classic triad of symptoms: gait and balance disorders, cognitive impairment, and urinary incontinence (2, 3). NPH, a specific type of communicating hydrocephalus, is classified into primary or idiopathic (iNPH) and secondary (sNPH) (4). Secondary NPH can occur at any age and is typically associated with specific causes such as subarachnoid hemorrhage, cerebral hemorrhage, encephalitis, craniocerebral trauma, etc. It is generally easier to diagnose due to its clear causal relationship, with symptoms manifesting after the primary disease. However, iNPH is more prevalent among the elderly. The nonspecific nature of its triad of symptoms makes differential diagnosis challenging, as these symptoms can overlap with those of other age-related conditions. Additionally, imaging findings of iNPH may be confounded by brain atrophy, leading to potential misdiagnosis as neurodegenerative diseases such as Alzheimer’s disease (AD) (5). Therefore, distinguishing iNPH from other neurological disorders remains a diagnostic challenge (6). In Norway, epidemiological studies on NPH reported an annual incidence of 5.5 per 100,000 population and a prevalence exceeding 21.9 per 100,000 population (7). However, there are reasons to suspect that these rates may be underestimated. First, the primary challenge lies in differentiating NPH from similar diseases. Second, there is evidence indicating a notable age-related increase in NPH incidence (8). Given the global rise in life expectancy, particularly over the past two decades, it is anticipated that the number of elderly patients with NPH will increase (9, 10). In recent years, the emergence of NPH as a significant public health challenge has driven a surge in research interest, reflected by a notable increase in the number of research and review articles on this topic. However, comprehensive reviews of NPH are currently unavailable. There are some reviews articles on NPH in the past, including epidemiological studies of NPH (11, 12), surgical treatment for NPH (13, 14), and pathogenesis and pathophysiology of NPH (15, 16). While these studies offer preliminary insight into the field of NPH, a comprehensive scientometric analysis of NPH is not available in literature.

Bibliometric analysis is an emerging tool that, unlike traditional literature reviews, focuses on quantifying literature, publications, authors, institutions, funding, and keywords (17). It utilizes statistical methods and visualization to explore the structure and trends of a subject or domain, enabling rapid analysis of publication patterns, characteristics, and interrelationships (18). Despite methodological limitations, bibliometrics is a valuable tool for evaluating scientific research within a specific subject or field. Therefore, bibliometric analysis has been extensively utilized in medical fields including neurology (19), neurosurgery (20), and oncology (21).

Therefore, we aimed to propose a scientometric approach for NPH and provide a comprehensive overview of the advancements in NPH research. Our objective was to explore the trending research fields in NPH and identify current hotspots. Additionally, we conducted detailed discussions on significant subtopics identified through bibliometric analysis. This study aims to assist both novice researchers and specialists in understanding the breadth of research topics, identifying new areas of interest, and shaping future research directions in the field of NPH.

## Methods

### Data source and retrieval strategy

We utilized the Web of Science Core Collection (WoSCC) to extract relevant literature on NPH published from January 2003 to December 2023. The data source edition was limited to the Web of Science Citation Index Expanded (SCI-Expanded) to ensure the high quality and authority of the included studies. All literature searches were performed on a single day (March 21, 2024) to minimize the impact of database updates on the number of retrieved documents. The search strategy was Topic = (“Normal Pressure Hydrocephalus” or “NPH”). We limited the literature types to articles and reviews, and restricted the language to English.

### Data extraction and collection

The literature was independently searched and screened by two researchers (TC and XH), and the results of both searches were then compared. Discrepancies in opinion were resolved by discussion with the third researcher (JW) and the optimal outcome chosen.

**Figure 1:**
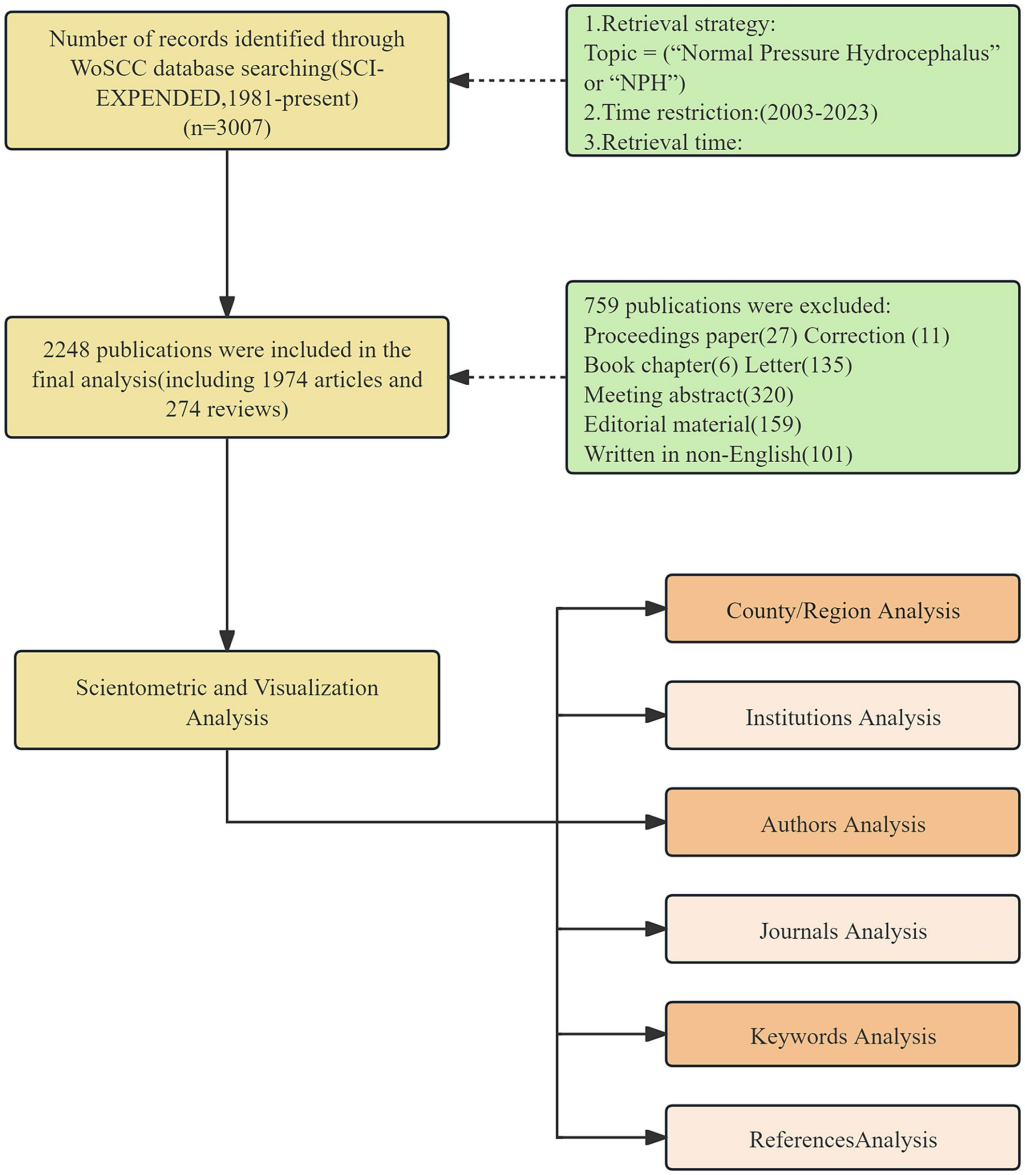
The flow chart of the included publications and methods used in the bibliometric analysis.

Based on the search strategies and restrictions outlined above, a total of 2,248 studies were found. The eligible publications were saved as plain text files and exported. Subsequently, the bibliographic records of these articles were imported into statistical software for analysis. Additionally, other information, such as the total number of citations, average citations per item (ACI), *H*-index, and Journal Impact Factor (IF), was obtained using the “Create Citation Report” function and Journal Citation Reports (JCR). Figure 1 illustrates the steps of the literature search and selection process.

### Data analysis and network mapping

We employed Microsoft Excel 2019 to analyze the annual number of published documents and citations. We utilized VOSviewer 1.6.18, developed by Leiden University in the Netherlands (22), to analyze the distribution and collaboration of countries or regions, institutions, authors and keywords. We employed CiteSpace 6.3, developed by Chen et al. (23), to calculate keywords burst, dual graph overlay of journals, and reference co-citation analysis. The visual graphs were generated using the aforementioned software tools, and the layout was refined using Pajek and SCImago Graphica for drawing visual maps and analyzing the research status, hotspots, and trends in NPH. In visualization maps, each node is depicted as a labeled circle. The size of each node is proportional to its frequency in the co-occurrence analysis. The color of each circle corresponds to the cluster to which it belongs. The thickness of the lines between nodes represents the strength of the connection and relevance between them.

### Research ethics

The data were obtained from public databases and did not involve human or animal participants. Therefore, there were no ethical considerations associated with the use of this data. No Ethics Committee approval was necessary.

## Results

### Scientific output

Using the described search strategy and selection process, a total of 2,248 related documents were identified, comprising 1,974 original articles and 274 reviews published between 2003 and 2023. The total number of citations for all publications was 53,710, with an average of approximately 23.89 citations per document. The *H*-index of the literature was determined to be 94.

**Figure 2:**
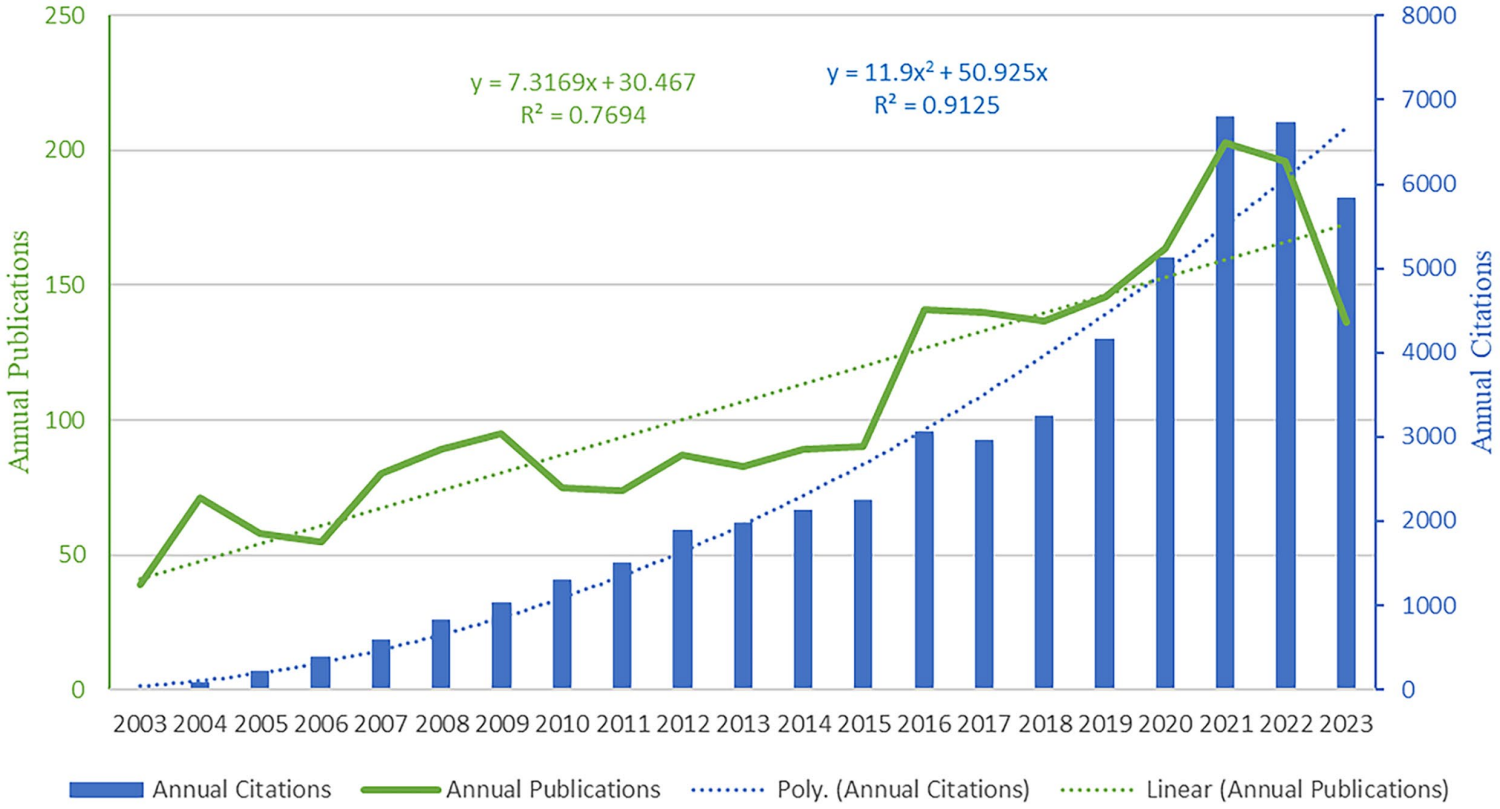
Distribution of the annual published documents and citations on HPH research from 2003 to 2023.

The changing trends in annual publications and citations of NPH research are depicted in Figure 2. Our analysis identified a significant increasing trend in the annual number of scientific publications over the past 20 years, with an *R*^2^ value of 0.7694. The number of publications increased from 39 in 2003 to 196 in 2022, representing a 402.56% increase. Regarding the citation number, the chart illustrates a similar increasing trend as observed in the annual publication number, with an R^2^ value of 0.99. Based on these results, it is evident that the interest in NPH has significantly increased in recent years, as indicated by the substantial growth in annual publication volume and citations.

### Analysis of countries and regions

**Figure 3:**
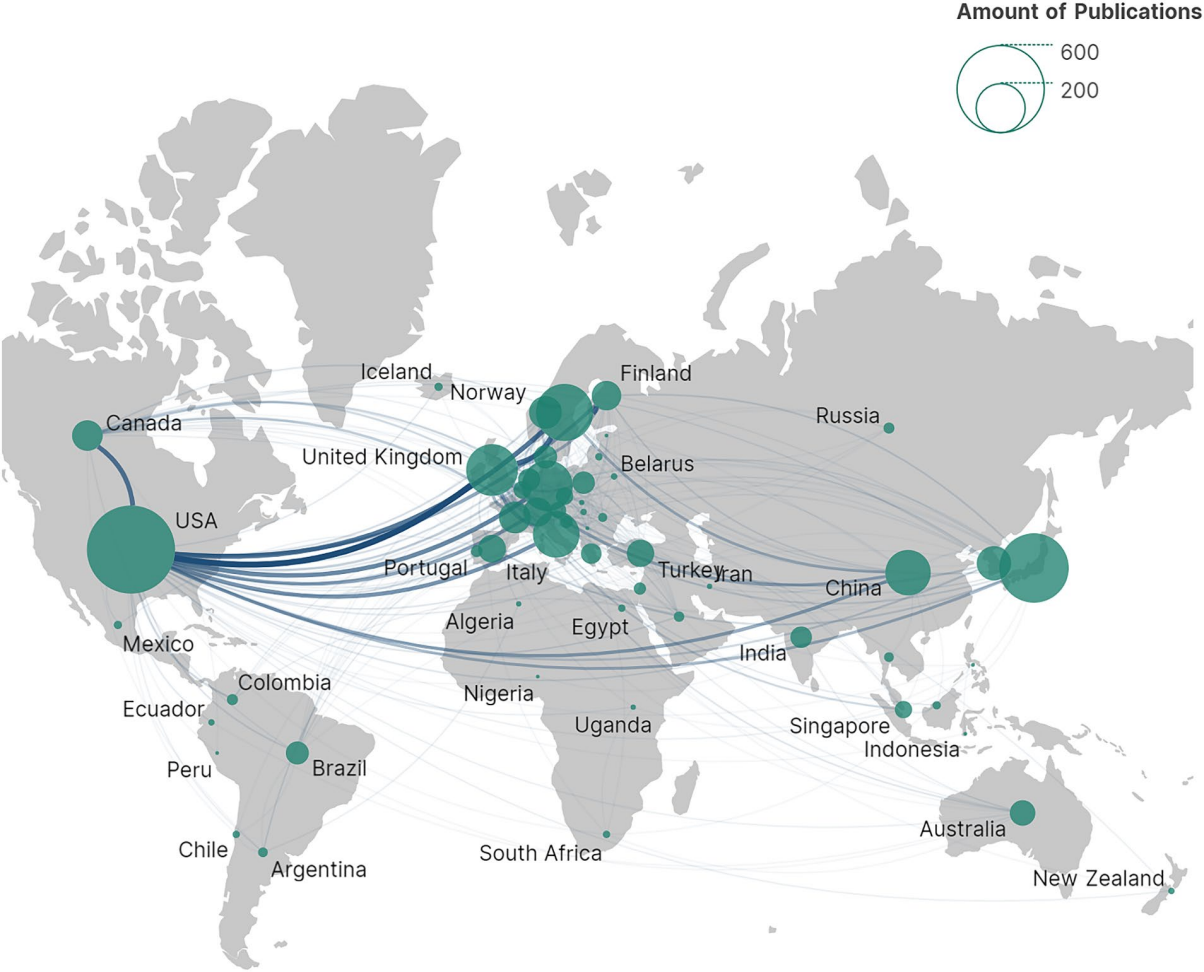
Distribution of countries/regions.

NPH is a global health challenge, with contributions from a total of 61 countries/regions to NPH research, as depicted in Figure 3. The data illustrates the worldwide distribution of publications, highlighting a concentration of publications in economically developed countries in Europe and the United States.

Table 1 presents the top 10 most productive countries. Regarding national research strength, the USA led with 573 publications and 18,472 citations, followed by Japan with 356 publications and 7,639 citations, and Sweden with 240 publications and 8,393 citations. The *H*-index is a dominant metric used to quantify the productivity and impact of authors, countries, or institutions. In terms of the *H*-index, the United States maintained the top position with an *H*-index of 69, followed by Sweden and Japan, both with *H*-indices above 40 (Table 1). Our study found that the United States led in both the total number of publications and *H*-index. Therefore, in terms of both research quantity and quality, the United States dominated in this research field. Additionally, it is noteworthy that although some countries had fewer publications, they obtained a high average number of citations per item (ACI). For example, Norway ranked highest in terms of ACI (average citations per item) at 40.16, which could be attributed to the publication of highly influential studies.

**Table 1:** Publications in the 10 most productive countries/regions.

Rank	Country/region	Count *n* (%)	Citations	*H*-index	ACI
1	USA	573 (25.489)	18,472	69	32.24
2	Japan	356 (15.836)	7,639	44	21.46
3	Sweden	240 (10.676)	8,393	48	34.97
4	England	194 (8.452)	5,998	40	30.92
5	Germany	190 (7.117)	5,531	37	29.11
6	Italy	160 (7.117)	3,028	28	18.93
7	China	123 (5.472)	1,020	15	8.29
8	South Korea	87 (3.870)	1,048	17	12.05
9	Norway	79 (3.514)	3,173	31	40.16
10	France	74 (3.292)	2,016	23	27.24

**Figure 4:**
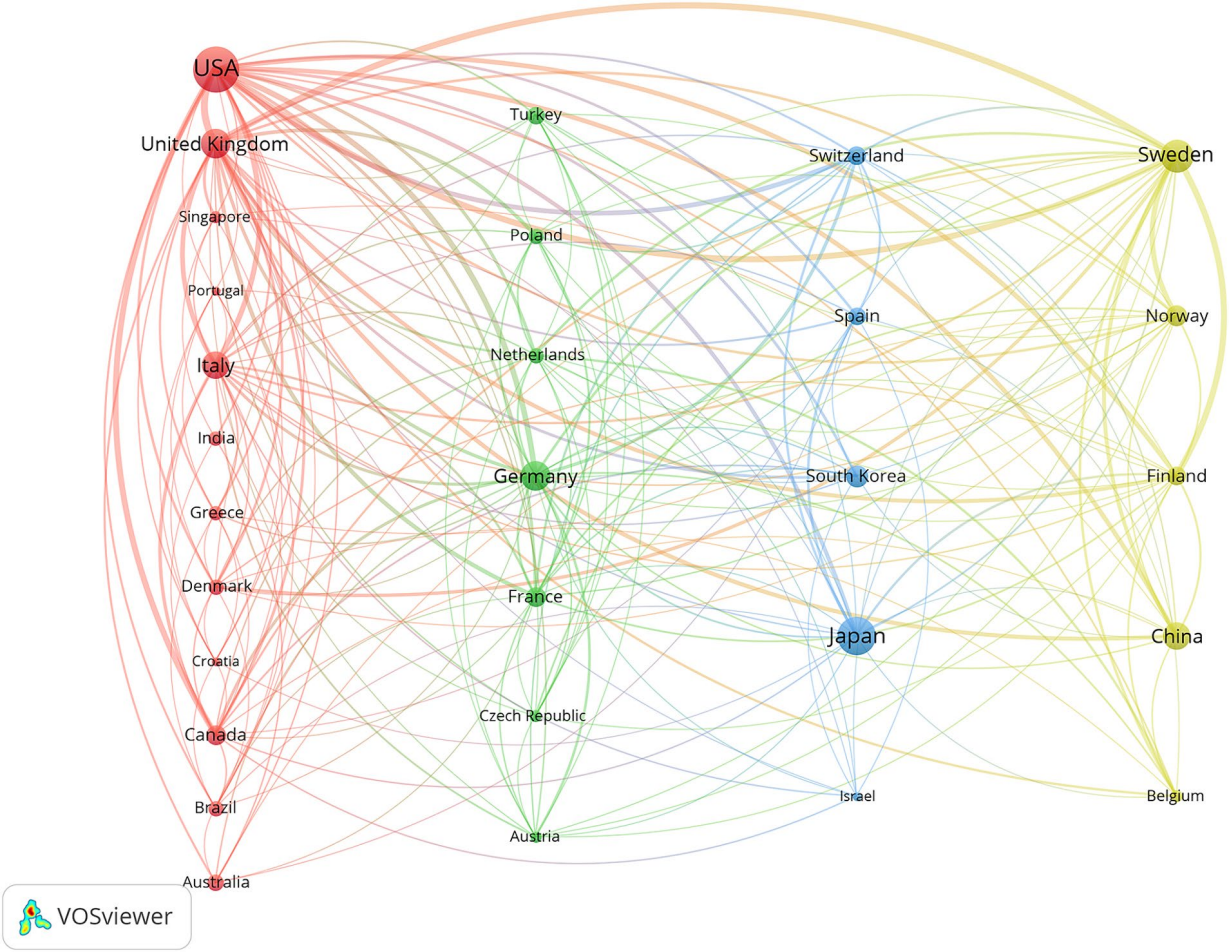
The co-authorship network of countries/regions.

VOSviewer was used for co-authorship analysis of the countries/regions to reveal international collaborations in NPH field. Figure 4 displays international cooperation among relevant countries/regions and only countries/regions with more than 10 papers were included. Of the 29 countries/regions that met this threshold. The 29 countries/regions were divided into 4 clusters represented by different colors. The largest cluster (in red), consisting of 12 countries, centered on the United States, United Kingdom and Italy. The USA had the most significant number of cooperating partners (*n* = 21). For NPH research, there is close cooperation between countries, highlighting NPH as a global challenge.

VOSviewer was utilized to conduct co-authorship analysis of countries/regions, revealing international collaborations in the field of NPH. Figure 4 illustrates international cooperation among relevant countries/regions, including only those with more than 10 papers. Among the 29 countries/regions that met this threshold, these countries/regions were divided into 4 clusters represented by different colors. The largest cluster (highlighted in red) consisted of 12 countries, centered around the United States, United Kingdom, and Italy. The USA had the highest number of cooperating partners (*n* = 21). In NPH research, close cooperation between countries highlights NPH as a global challenge.

### Analysis of institutions

In the analysis of institutions, a total of 2,132 institutions contributed to the published research in the field. The top 10 most prolific institutions are listed in Table 2. Among the top 10 institutions, eight were from Europe, one was from the United States, and one was Asian. Among the most productive institutions, the University of Gothenburg had 108 publications and 3,811 citations, followed by Umea University with 80 publications and 2,065 citations, and the University of Oslo with 73 publications and 3,082 citations. As shown in Table 2, the University of Gothenburg had the highest *H*-index value (*n* = 36), followed by the University of Oslo (*n* = 30) and the National Hospital Norway (*n* = 28). Regarding ACI, the top three institutions with the largest ACI were the National Hospital Norway (*n* = 42.59 times), followed by the University of Oslo (*n* = 42.22 times), both of which are from Norway.

**Table 2:** Publications in the 10 most productive institutions.

Rank	Institution	Country	Count *n* (%)	Citations	*H*-index	ACI
1	University of Gothenburg	Sweden	108 (4.806)	3,811	36	35.29
2	Umea University	Sweden	80 (3.560)	2,065	27	25.81
3	University of Oslo	Norway	73 (3.249)	3,082	30	42.22
4	Juntendo University	Japan	68 (3.026)	2,224	23	32.71
5	University of London	Britain	65 (2.893)	1,970	21	30.31
6	National Hospital Norway	Norway	64 (2.848)	2,726	28	42.59
7	Johns Hopkins University	USA	63 (2.804)	1,717	23	27.25
8	Kuopio University Hospital	Finland	61 (2.715)	2,106	22	34.52
9	University of Cambridge	Britain	61 (2.715)	1,884	22	30.89
10	University of Eastern Finland	Finland	61 (2.715)	2,058	22	33.74

The University of Gothenburg’s most frequent collaborator is Sahlgrenska University Hospital, with 51 joint publications. The university’s most cited article, “Prevalence of Idiopathic Normal-Pressure Hydrocephalus,” was published in 2014. This study investigates the prevalence of normal pressure hydrocephalus among adults aged 70 years and older, providing robust epidemiological data on this condition in an aging population. The most prolific author affiliated with the University of Gothenburg in this field is M. Tullberg, who has authored 42 articles on normal pressure hydrocephalus, ranking 9th among the top 10 authors in terms of total publications.

**Figure 5:**
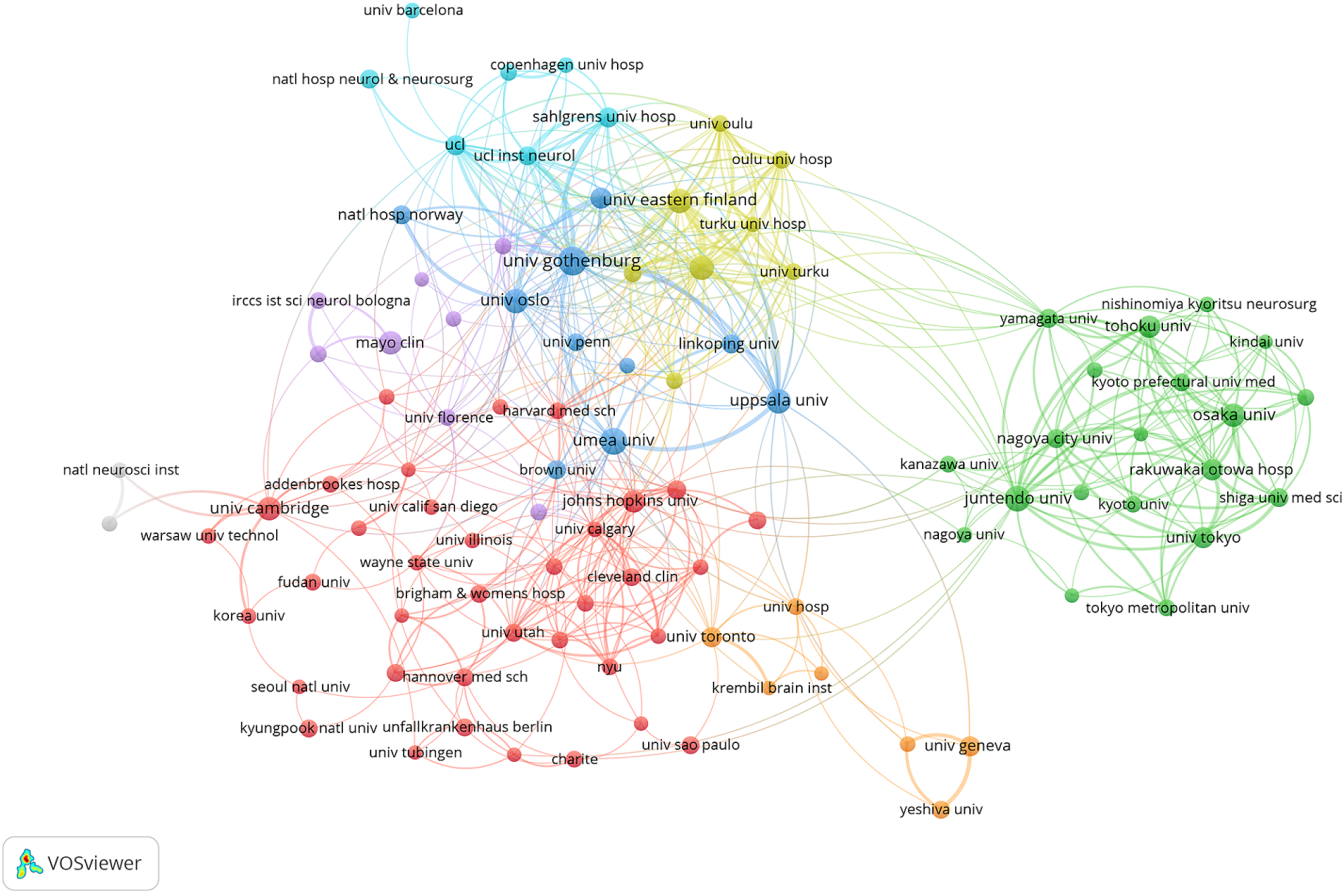
The co-authorship network of institutions.

VOSviewer was used to create the network visualization map depicting institutions’ cooperation (Figure 5). With a minimum threshold of 10 articles published by institutions, 100 institutions met the criteria. There were seven clusters of co-authorship represented by different colors. The largest cluster, highlighted in red, consisted of 37 institutions centered around the University of Cambridge, Johns Hopkins University, and the Cleveland Clinic. The map shows that there is a certain amount of collaboration and exchange between research institutions around the world, but mostly restricted either to a country or a particular region.

### Analysis of authors

In total, 8,363 authors are involved in research related to NPH, and Table 3 lists the top 10 authors. In terms of publications, Eide P. K. has published 63 articles and had 2,613 citations, followed by Leinonen V. with 59 publications and 2,080 citations, and Miyajima M. with 53 publications and 1,759 citations. The author with the highest ACI was Ishikawa M. (*n* = 46.41), followed by Eide P. K. (*n* = 41.48) and Arai H. (*n* = 36.1).

**Table 3:** Publications in the 10 most productive authors.

Rank	Author	Count *n* (%)	Citations	*H*-index	ACI
1	Eide P. K.	63 (2.804)	2,613	27	41.48
2	Leinonen V.	59 (2.626)	2,080	22	35.25
3	Miyajima M.	53 (2.359)	1,759	21	33.19
4	Malm J.	50 (2.225)	1,279	22	25.58
5	Eklund A.	49 (2.181)	1,225	22	25
6	Czosnyka M.	47 (2.092)	1,330	18	28.3
7	Ishikawa M.	44 (1.958)	2,042	21	46.41
8	Yamada S.	43 (1.914)	518	17	18
9	Tullberg M.	42 (1.869)	1,207	22	28.74
10	Arai H.	41 (1.825)	1,480	20	36.1

In our analysis of Eide P. K.’s articles, we discovered that his research on normal pressure hydrocephalus (NPH) is comprehensive, covering epidemiology, pathogenesis, and surgical treatment, with a particular focus on the role of the glymphatic system in the pathogenesis of NPH. Notably, a 2017 paper he co-authored, titled “Glymphatic MRI in Idiopathic Normal Pressure Hydrocephalus,” has been cited 400 times. This landmark study was the first to evaluate glymphatic system function in NPH patients using magnetic resonance imaging.

Closely following is Leinonen V., who, along with his team, focused their research on biomarkers for the diagnosis and prognosis of NPH. Their representative article, “Cerebrospinal Fluid Biomarker and Brain Biopsy Findings in Idiopathic Normal Pressure Hydrocephalus,” is a notable contribution to the field. Although Eide P. K. and Leinonen V. have both published extensively on NPH, they have only collaborated twice. This suggests that most studies in this field are conducted independently rather than through the collaborative efforts of the authors listed in Table 3.

**Figure 6:**
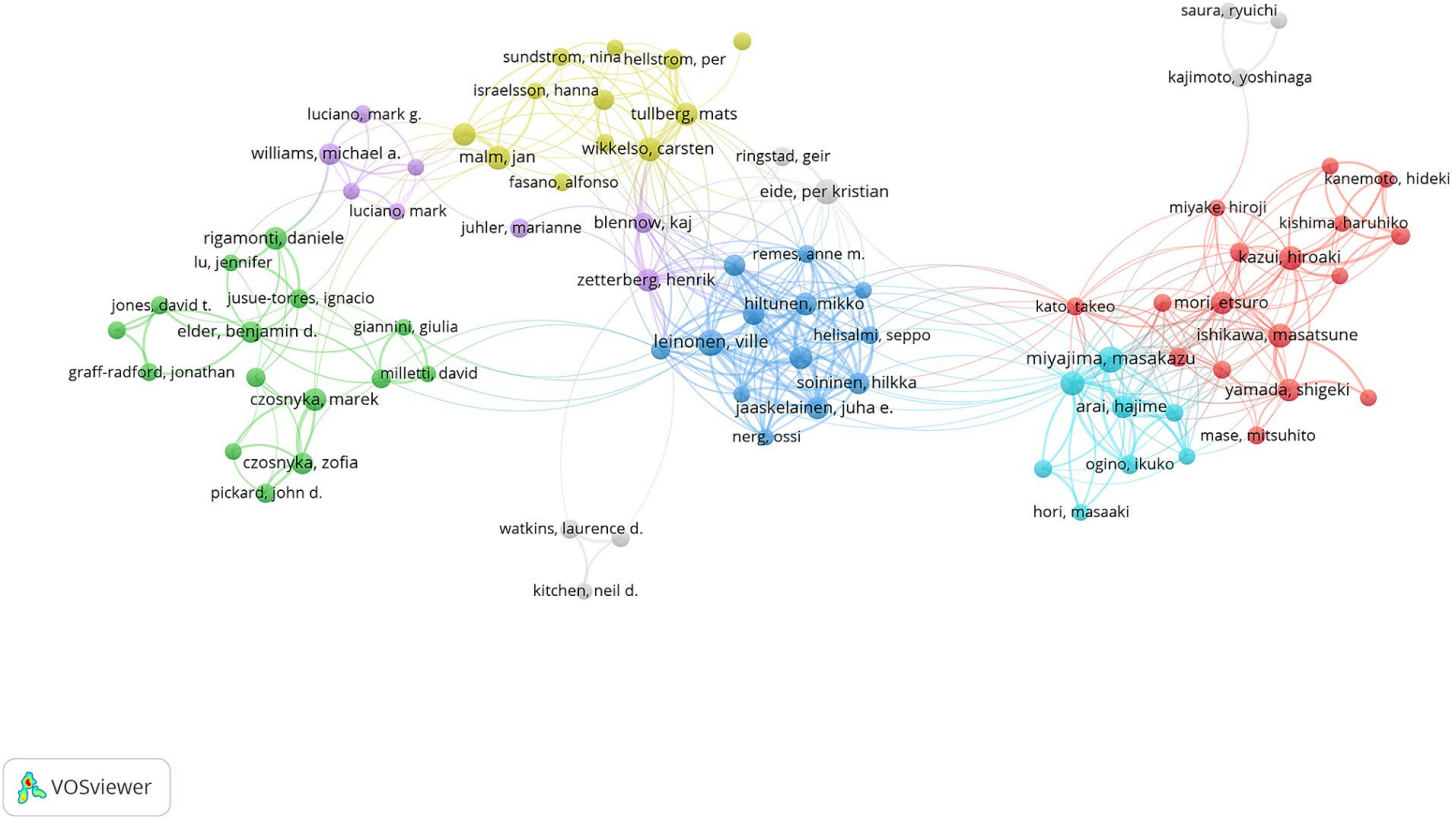
The co-authorship network of authors.

We used VOSviewer to perform co-authorship analysis, including only authors with a minimum of 10 publications (Figure 6). Co-authorship networks involved 93 authors and were divided into six color-coded clusters. The largest cluster, represented in red and consisting of 17 authors, stood out prominently. The majority of cluster members are native authors; therefore, international research teams should improve their communication.

### Analysis of journals

Relevant articles were published in 510 journals. The Journal of Neurosurgery (101 publications, 4.495% of total) ranks first, followed by Acta Neurochirurgica (96 publications, 4.272%) and World Neurosurgery (80 publications, 3.560%). All of the top 10 journals belong to the field of neurology. With the exception of Clinical Neurology and Neurosurgery, these journals all have a 2023 impact factor greater than 2. Fluids and Barriers of the CNS has the highest impact factor (*n* = 7.3).

**Figure 7:**
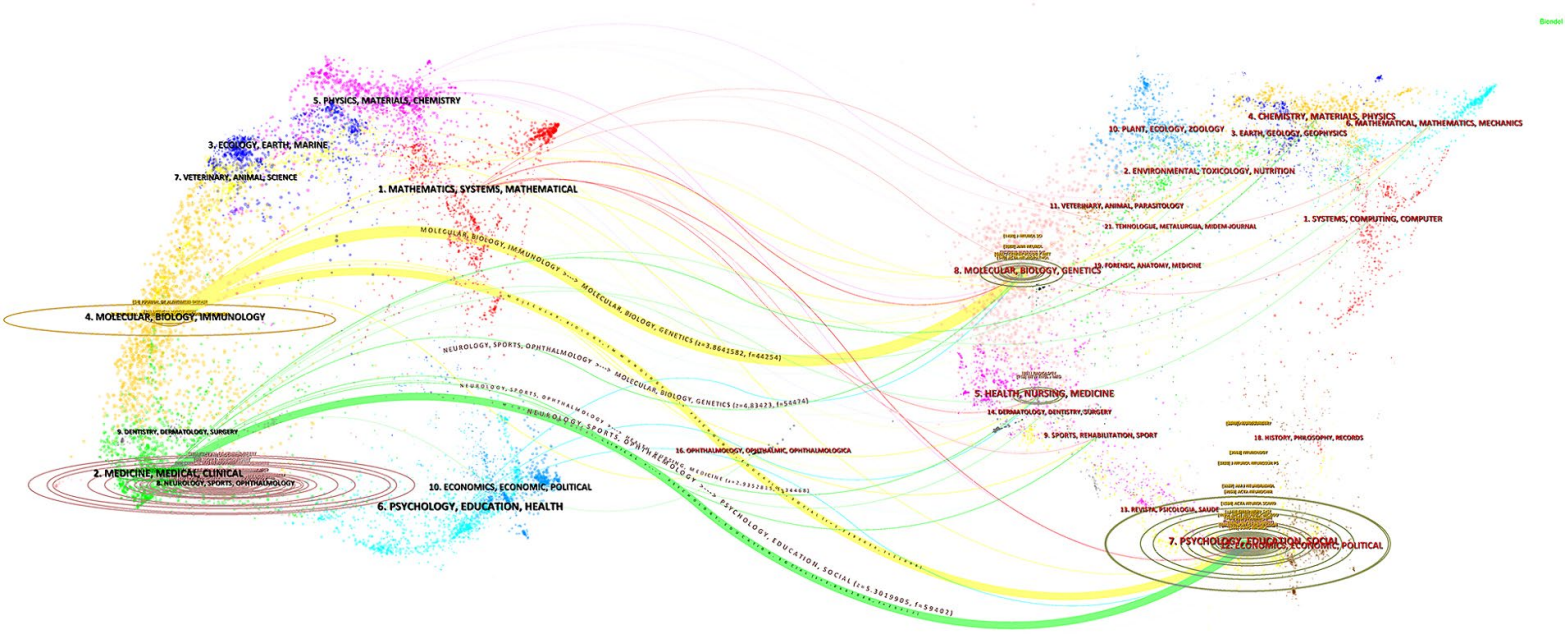
The dual-map overlay of journals.

Dual-map overlays allowed us to accomplish several innovative visual analytic tasks that were previously not possible to perform intuitively. By following the citation arcs from the origin branch to the concentrated landing zones, it became straightforward to determine whether a set of publications incorporated prior work from multiple disciplines (24). We conducted an analysis of published and co-cited journals using a journal dual-map overlay, depicted in Figure 7. The citing journals are represented on the left, and the cited journals are represented on the right. Citation relationships are depicted as colored lines from left to right. We identified two distinct citation paths represented by orange and green lines. The green path is an independent development pattern, the citing journals mainly belong to Molecular Biology/Clinics, and cited journals mainly belong to Psychology/Education/Social. Whereas, the orange pathway is a confluent pattern of development, indicating that the two crosscutting areas evolved into a common research theme, the citing journals mainly belong to Molecular Biology/Immunology, and cited journals mainly belong to Molecular Biology/Clinics and Psychology/Education/Social.

### Analysis of co-occurring keywords and burst terms

Indexing keywords facilitates understanding the main content of a paper, so to a certain extent, keyword analysis illustrates the hotspots and focus of this research area. Before analyzing, we remove to the search terms, and we merged some terms with the same meaning, (e.g., “csf” and “cerebrospinal fluid,” “Alzheimer’s disease” and “Alzheimer disease,” “biomarker” and “biomarkers”). In this study, a comprehensive analysis was conducted on 6,334 extracted keywords, and the top 10 frequently occurring keywords are “Cerebrospinal fluid,” “diagnosis,” “management,” “Alzheimer’s-disease,” “dementia,” “ventriculoperitoneal shunt,” “magnetic resonance imaging,” “brain,” “disease,” “shunt surgery.”

**Figure 8:**
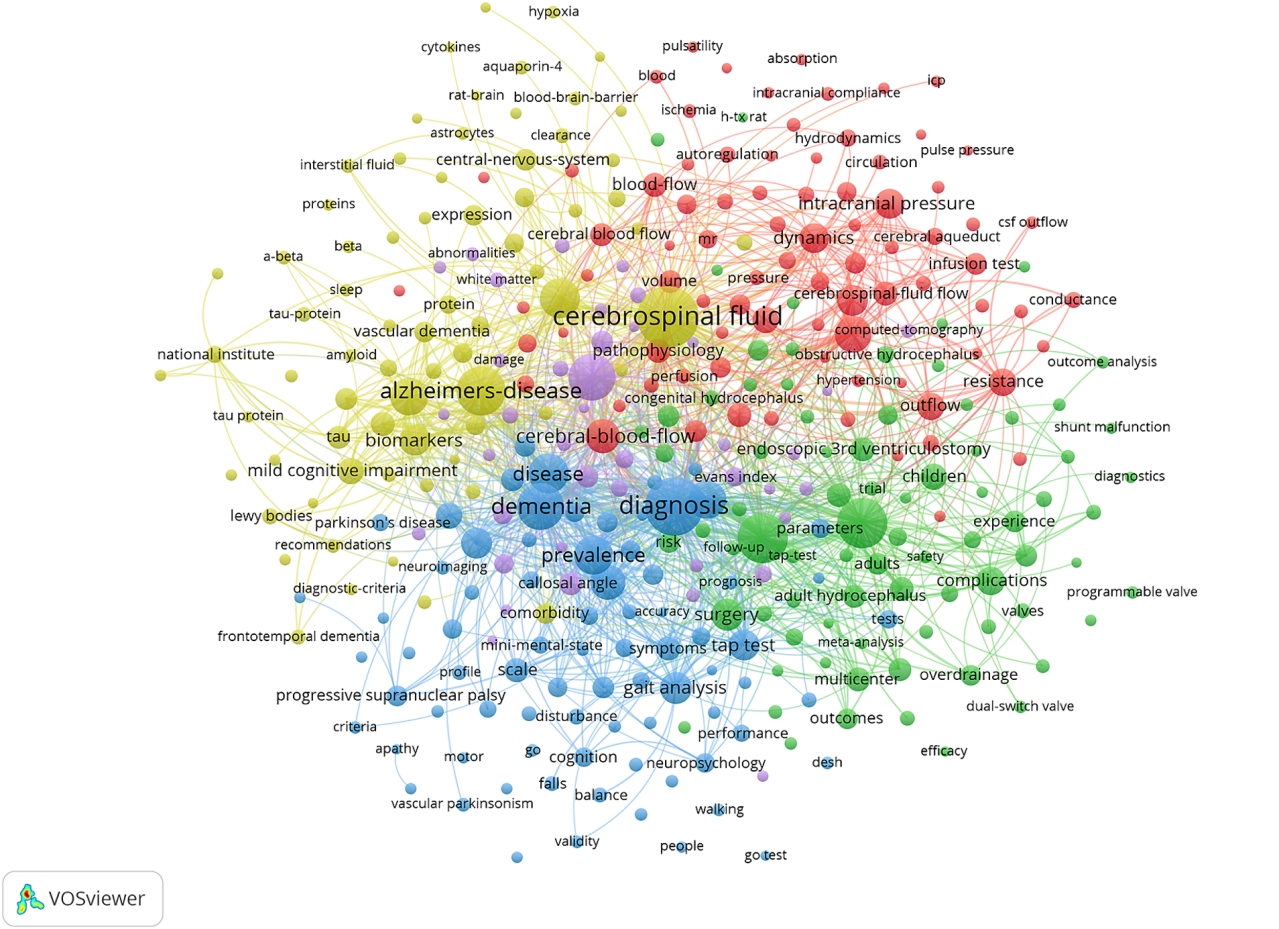
Keyword co-occurrence networks.

In the keyword co-occurrence network (Figure 8), the keywords were assigned to 5 clusters according to the color: cluster 1 (red) included keywords related to pathogenesis and pathophysiology, such as “intracranial pressure,” “blood flow,” “dynamics,” etc. Cluster 2 (green) included keywords related to treatment, such as “ventriculoperitoneal shunt,” “endoscopic 3rd ventriculostomy,” and “shunt.” Cluster 3 (blue) included keywords related to diagnosis, such as “callosal angle,” “dementia.” Cluster 4 (yellow) included keywords related to differential diagnosis such as “Alzheimer disease,” “biomarkers,” and “proteins.”

**Figure 9:**
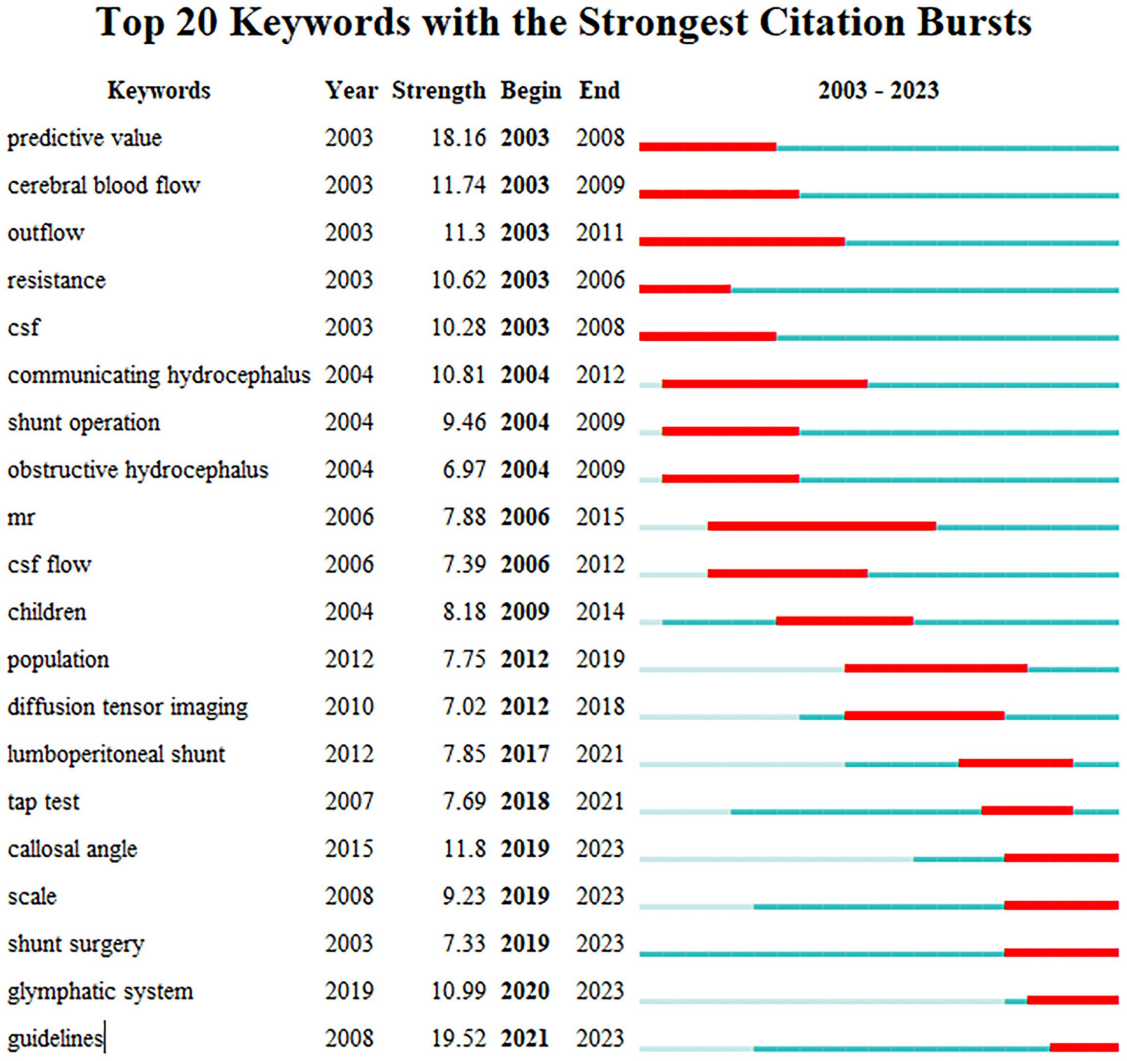
The top keywords with the strongest citation bursts. The long blue line depicts the timeline (2003–2023), and the short red line indicates the burst period of certain keyword.

Burstness highlights the period when the occurrence or use of specific nodes is significantly higher compared to other nodes. Specifically, when there is a sudden spike in publications on a particular subject or area of interest, a keyword burst can reveal new developments related to that field. Furthermore, burstness indicates nodes that have garnered significant attention in a short period, showing substantial changes in frequency over a brief timeframe. We used CiteSpace software to extract citation bursts for all keywords, specifically focusing on the top 20 keywords (Figure 9). The red timeline in Figure 9 illustrates the duration of the outbreak. Based on the development trends shown in the Figure 9, the study period can be divided into three phases: 2003–2009, 2010–2018, and 2019–2023. The strongest bursts in 2003–2009 included the following keywords: “predictive value,” “cerebral blood flow,” “resistance,” “CSF,” “communicating hydrocephalus,” “shunt operation,” obstructive hydrocephalus.” Studies in this period largely focused on the pathogenesis and differential diagnosis of normal pressure hydrocephalus. Regarding pathogenesis, researchers concentrated on cerebrospinal fluid circulation disorders and intracerebral microcirculation and vascular dysfunction. The period from 2010 to 2018 recorded the keywords with the strongest citation bursts: “MRI” and “diffusion tensor imaging.” During this time, research primarily began utilizing imaging technologies to explore the pathogenesis of normal pressure hydrocephalus (NPH) and identify potential imaging biomarkers. The period from 2016 to 2021 shows five keywords that have recently received significant attention: “callosal angle,” “scale,” “shunt surgery,” “glymphatic system,” and “guidelines.” The most notable keyword, “guidelines,” attained the highest burst strength during this period. This suggests that researchers worldwide are increasingly focusing on accurate diagnosis and treatment of NPH, and are eager to establish standardized guidelines.

### Analysis of co-cited references

A co-citation relationship is established when two studies are concurrently cited by a third study (24). Co-citation analysis further illustrates the frequency with which articles are cited together. This analysis of co-cited works helps constitutes the theoretical basis and knowledge framework of a scientific research topic. We identified the top 10 co-cited references in Table 4. The most frequently cited reference is the guidelines for management of INPH (third edition), and the second version of the guide is also cited at fifth. The second article primarily addresses the prevalence of iNPH, which is related to the topic of the seventh article. The third article discusses the role of the glymphatic system in the pathogenesis of NPH.

**Table 4:** The top 10 co-cited references.

Rank	Citation counts	Author	Reference title	Journal	Year
1	109	Nakajima M.	Guidelines for management of idiopathic normal pressure hydrocephalus (third edition): endorsed by the Japanese Society of normal pressure hydrocephalus	Neurol Med Chir	2021
2	82	Jaraj D.	Prevalence of idiopathic normal-pressure hydrocephalus	Neurology	2014
3	80	Ringstad G.	Glymphatic MRI in idiopathic normal pressure hydrocephalus	Brain	2017
4	74	Kazui H.	Lumboperitoneal shunt surgery for idiopathic normal pressure hydrocephalus (SINPHONI-2): an open-label randomized trial	Lancet Neurol	2015
5	70	Mori E.	Guidelines for management of idiopathic normal pressure hydrocephalus: second edition	Neurol Med Chir	2012
6	63	Espay A. J.	Deconstructing normal pressure hydrocephalus: ventriculomegaly as early sign of neurodegeneration	Ann Neurol	2017
7	60	Andersson J.	Prevalence of idiopathic normal pressure hydrocephalus: a prospective, population-based study	PLoS One	2019
8	58	Giordan E.	Outcomes and complications of different surgical treatments for idiopathic normal pressure hydrocephalus: a systematic review and meta-analysis	J Neurosurg	2019
9	53	Williams Michael A.	Diagnosis and treatment of idiopathic normal pressure hydrocephalus	Continuum	2016
10	51	Kockum K.	The idiopathic normal-pressure hydrocephalus Radscale: a radiological scale for structured evaluation	Eur J Neurol	2018

**Figure 10:**
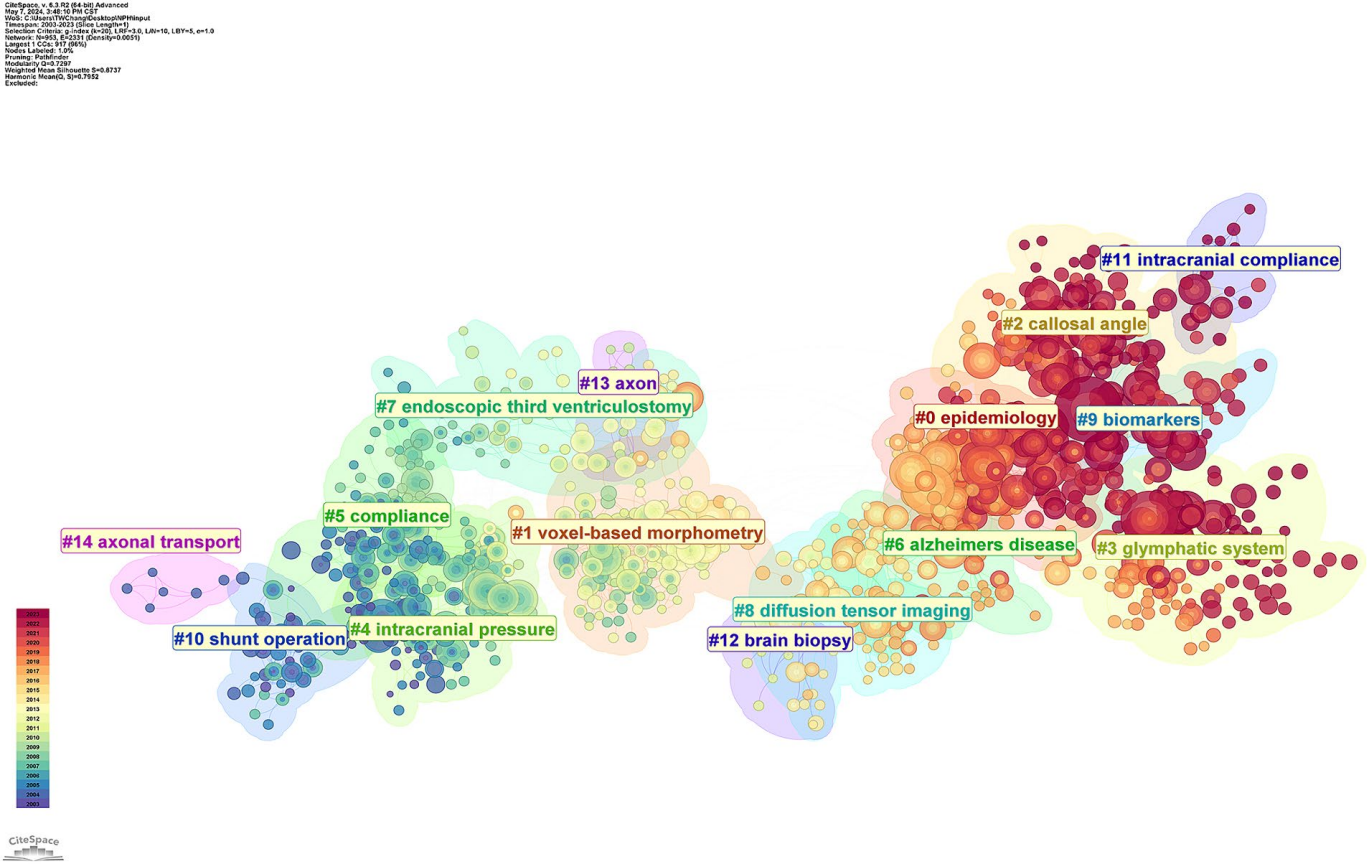
CiteSpace visualization clusters of the co-cited references. Terms from the title field of the citing papers within each cluster are used as the definition of that cluster.

The co-citation network can be segmented into distinct clusters using CiteSpace. Papers within the same cluster are closely related based on their co-citation patterns. Each cluster is defined by terms extracted from the keywords field of the referenced documents in that cluster. We can find the top 14 clusters, which are #0 epidemiology, #1 voxel-based morphometry, #2 shunt operation, #3 intracranial compliance #4 brain biopsy, #5 axon, #6 axonal transport, #7 callosal angle, #8 glymphatic system, #9 intracranial pressure, #10 compliance, #11 Alzheimer’s disease, #12 endoscopic third ventriculostomy, #13 diffusion tensor imaging, and #14 biomarkers (Figure 10). Eight of these clusters (#3, #4, #5, #4, #6, #8, #6, and #10) are about the pathogenesis of NPH, and three of these clusters (#1, #7, and #13) focus on imaging study of NPH. Whereas the other two (#2 and #12) focus on surgical treatment of NPH.

**Figure 11:**
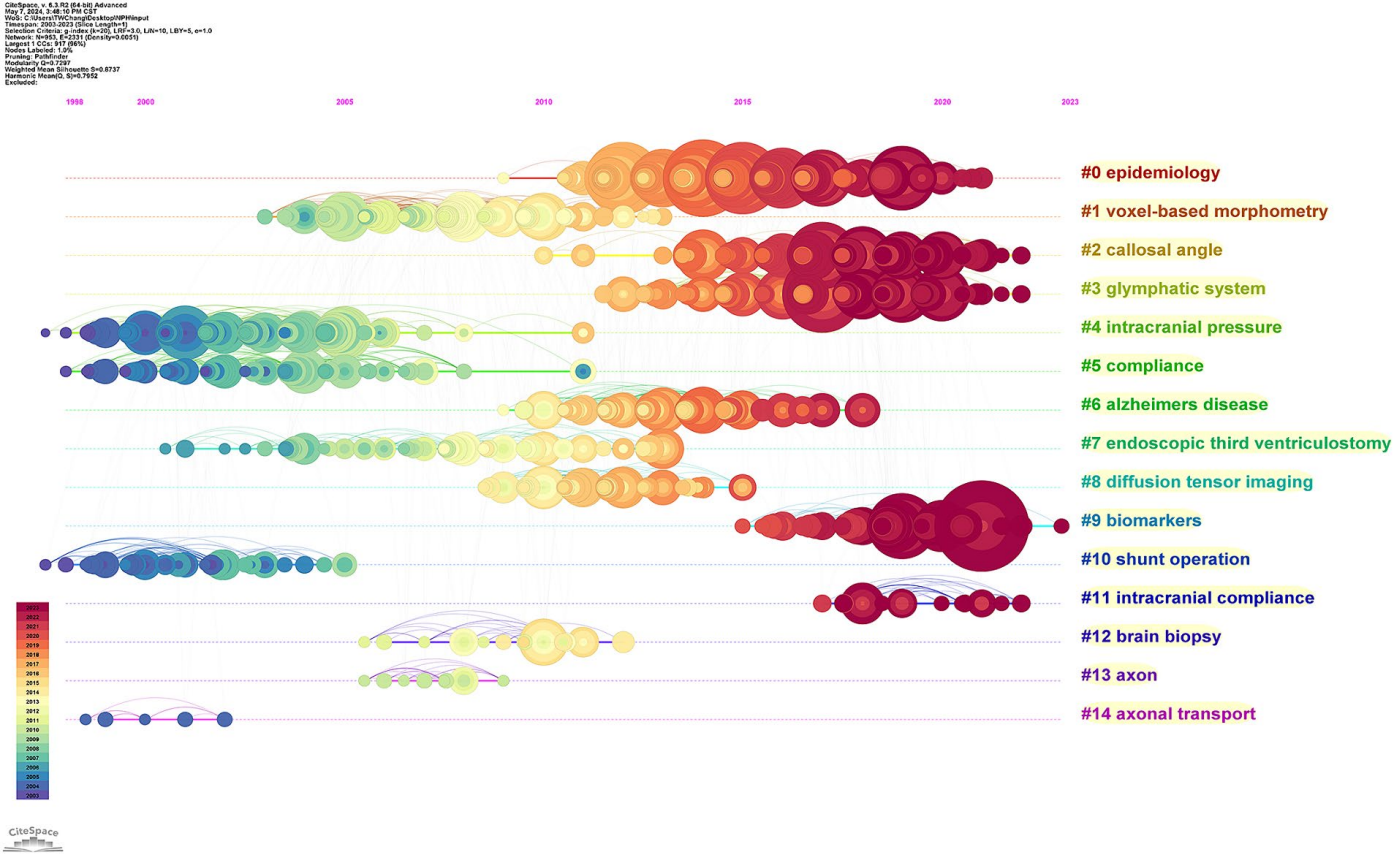
Timeline view of these listed clusters of the co-cited references.

The cluster map can be transformed into a timeline diagram to analyze research evolution and development within the listed clusters over time. Early studies primarily focused on the surgical treatment and pathogenesis of normal pressure hydrocephalus (Figure 11). At the same time, we can also observe that the research on the pathogenesis continues to the present, and the main concern is the role of the intracranial lymphatic system in the pathogenesis. Another current area of interest is to identify biomarkers that can accurately diagnose and predict the prognosis of normal pressure hydrocephalus.

## Discussion

### Global trends in NPH research

Research on normal pressure hydrocephalus (NPH) has entered a phase of rapid development. Research on NPH has shown consistent growth over the past two decades, as shown in Figure 2. NPH has emerged as a compelling topic across multiple disciplines and is anticipated to continue gaining momentum in the future. This trend can be attributed to the development of the economy and society, as developed countries continue to experience population aging. As the population ages, the incidence of normal pressure hydrocephalus continues to rise. Consequently, an increasing number of researchers are focusing on this field.

Research initiatives in the field of NPH have been initiated in several countries, with the United States leading the way. A total of 1,573 publications originated from the USA, representing approximately a quarter of all publications. Figure 4 illustrates extensive intercountry cooperation among countries/regions. The USA emerged as the most productive country and the hub of international cooperation. Therefore, the USA led the forefront of scientific and academic research.

Among the top 10 most productive institutes, eight were European institutions, one was Asian, and one was American. European institutions led in terms of quantity of NPH research. Figure 6 illustrates that there is a certain amount of collaboration and exchange between research institutions around the world, but mostly restricted either to a country or a particular region.

Additionally, the top three most prolific authors in this field are Eide P. K., Leinonen V., and Miyajima M. They are leaders in the field and are likely to continue shaping its development. These top scholars are ideally suited for collaboration and communication.

Within this domain, there are specific journals that deserve researchers’ attention. The journals listed in Table 5, such as Journal of Neurosurgery, Acta Neurochirurgica, and World Neurosurgery, are likely core journals in this field, making them recommended venues for submitting relevant papers. Researchers should also prioritize keeping up with the latest articles published in these journals.

**Table 5:** The top 10 most productive journals.

Rank	Journal	Counts (%)	Citations	WoS categories	IF (2023)	Quartile (2023)
1	Journal of Neurosurgery	101 (4.495)	2,545	Clinical Neurology	4.1	Q1
2	Acta Neurochirurgica	96 (4.272)	1,541	Clinical Neurology	2.4	Q3
3	World Neurosurgery	80 (3.560)	922	Clinical Neurology	2.0	Q4
4	Neurosurgery	73 (3.249)	3,469	Clinical Neurology	4.8	Q1
5	Clinical Neurology and Neurosurgery	69 (3.071)	1,006	Clinical Neurology	1.9	Q4
6	Fluids and Barriers of the CNS	56 (2.492)	731	Neurosciences	7.3	Q1
7	Journal of Alzheimers Disease	53 (2.359)	1,100	Neurosciences	4.0	Q2
8	Journal of the Neurological Sciences	47 (2.092)	950	Clinical Neurology	4.4	Q2
9	American Journal of Neuroradiology	45 (2.003)	1,574	Neuroimaging	3.5	Q2
10	Acta Neurologica Scandinavica	44 (1.958)	1,366	Clinical Neurology	3.5	Q2

### Hot spots and future research direction

Through keyword burst detection and cited references clustering analysis, research hotspots and current research directions may be discovered. Thus, combined with keywords and references that continue to show high bursts, we analyzed current research hotspots and predicted future research trends.

#### Epidemiology

The variations in diagnostic criteria, survey populations, age groups, gender distributions, and racial compositions employed across different studies pose challenges when comparing the epidemiological findings of iNPH. In a prevalence study conducted in Norway in 2008, using the diagnostic criteria outlined in the 2005 international guidelines, the reported prevalence rates were 21.9 per 100,000 individuals for “probably iNPH” and 29 per 100,000 individuals for “possible iNPH” within hospital settings.

A Swedish study revealed that among the general population, the prevalence rate of “probably iNPH” was 3.7%, with rates varying between 0.2% for individuals aged 70–79 years and 5.9% for those aged above 80, without significant gender disparity observed. However, an epidemiological survey in Sweden in 2019, using Japanese iNPH diagnostic criteria, indicated a lower prevalence rate of “probably iNPH,” at approximately 1.5% (25, 26). Another survey in Japan during 2008, employing Japanese diagnostic criteria, demonstrated a prevalence rate of “MRI-supported possible iNPH” at around 2.9%. A recent MRI-based study (27) focusing primarily on 70-year-olds in Sweden, using international guideline diagnostic criteria, reported a higher prevalence rate of approximately 1.5% for “possible iNPH,” significantly greater than the rate reported in 2019.

Future research efforts should focus on developing more authoritative diagnostic guidelines for NPH. Researchers can then conduct large-scale population studies involving multiple countries and research centers, based on these standardized guidelines. Such comprehensive studies will provide more accurate epidemiological data on the prevalence and incidence of NPH, which will be invaluable for clinical practitioners.

#### Glymphatic system

The glymphatic system is a brain fluid transport system composed of several key components. Current research indicates that the glymphatic system includes cerebrospinal fluid (CSF), perivascular spaces (PVS), aquaporin-4 (AQP4) channels located on astrocytic end feet, and interstitial fluid (ISF) (28). AQP4 plays a critical role in mediating the exchange of CSF and ISF within this system, facilitating the clearance of metabolic byproducts and toxic proteins in the central nervous system. This mechanism is vital for maintaining brain homeostasis and supporting overall brain health.

Cerebrospinal fluid (CSF) in the subarachnoid space flows into perivascular spaces surrounding arteries, and water within the CSF passes through aquaporin-4 (AQP4) channels located on astrocytic end feet into the brain parenchyma for cerebrospinal fluid-interstitial fluid (CSF-ISF) exchange. After this exchange, the interstitial fluid (ISF) containing significant metabolic waste enters AQP4 channels, flows into perivascular spaces around veins, and then gradually returns to the subarachnoid space before entering venous circulation, thereby constituting the glymphatic system (29).

PVS and AQP4 are two crucial structures involved in the glymphatic system with implications for various central nervous system diseases (30). PVS represent microscopic tissue spaces surrounding arteries and veins within the brain; their loose fibrous matrix provides low-resistance pathways for efficient CSF-ISF flow essential for intracranial fluid transport. Astrocytes possess orthogonal arrangements of AQP4 on their end feet which facilitate efficient water transportation. When AQP4 is polarly distributed on the outer wall of the PVS end feet, it facilitates the rapid exchange of CSF with ISF in the brain tissue, promoting the clearance of brain metabolic waste. Furthermore, the polar distribution of AQP4 is synchronized with the strength of the astrocytic lymphatic system. Therefore, structural damage to the PVS and abnormal polar distribution of AQP4 may be an important cause of impaired CSF-ISF exchange, metabolic waste deposition, and ultimately the development or exacerbation of neurological diseases.

Loss of arterial compliance can lead to glymphatic system damage in iNPH (16). In patients diagnosed with iNPH, the incidence of systemic atherosclerotic diseases, including primary hypertension, dyslipidemia, coronary artery disease, and peripheral arterial disease, is significantly higher (31). As the vessels become increasingly rigid, the weakening of vascular pulsatility and the diminished driving force for glymphatic fluid flow lead to the accumulation of waste in the brain interstitium, increasing the resistance to outflow along the lymphatic pathways, which is part of the reason why CSF retrograde into the ventricular system. The restoration of cerebrospinal fluid dynamics after shunting may promote glymphatic fluid flow and improve cognitive function.

The physiological function of aquaporins is to facilitate the transport of water across cell membranes. Reduced expression of AQP-4 has been confirmed as another potential mechanism for lymphatic vessel damage in iNPH patients (32). In Alzheimer’s disease, decreased expression of AQP-4 leads to impaired clearance of misfolded proteins, resulting in neurotoxicity and cognitive decline. Glymphatic system damage in iNPH may cause accumulation of metabolic waste, leading to neurotoxicity and cognitive impairment.

Impairment to the glymphatic system disrupts brain fluid dynamics and hinders the clearance of metabolic waste, critically contributing to the pathogenesis of NPH. Given these established mechanisms and targets, future research should prioritize investigating methods to ameliorate NPH symptoms and prognosis through modulation of glymphatic system function.

#### Biomarkers

Cerebrospinal fluid (CSF) biomarker testing can yield valuable biological information. There has been increasing interest in identifying appropriate biomarkers for diagnosing, differentiating diagnoses, and potentially predicting outcomes using CSF.

Total tau protein (t-tau) (33) is generally considered a non-specific marker of neuronal/axonal degeneration, while phospho-tau (p-tau) (34) reflects tau hyperphosphorylation as well as tangle formation. Aβ42 is indicative of the presence of amyloid pathology (35). Kapaki et al. (36) analyzing CSF biomarkers in iNPH, which showed that t-tau was significantly increased in iNPH, as compared with the control group, whereas Aβ42 was decreased. Ray et al. (37) reported different results that showed a significant decrease in CSF Aβ42 concentration in NPH patients as compared with the control group, but no significant difference in t-tau or p-tau between these two groups. Taghdiri et al. (38) showed that lower CSF Aβ42 and p-tau concentrations were observed in patients with iNPH compared to health controls.

The researchers proposed using cerebrospinal fluid biomarkers to distinguish diseases similar to iNPH, such as the most common Alzheimer’s disease. Taghdiri et al. (38) suggested that the concentration of Aβ42 in cerebrospinal fluid of patients with iNPH was higher than that of patients with AD, and the concentration of t-tau and p-tau was lower than that of patients with AD. Mazzeo et al. (39) agreed that Aβ42/Aβ40, p-tau, and t-tau were significantly lower in iNPH than AD, but found no significant difference in Aβ42 concentrations.

Meanwhile, researchers have started to investigate the potential correlation between biomarker concentrations and shunt responsiveness. Thavarajasingam et al. (40) compared two groups of patients with and without improvement in symptoms after shunt surgery, and found significantly higher levels of t-tau and p-tau in the cerebrospinal fluid of iNPH patients who did not experience symptom improvement post-shunting, as compared to those who did. However, Tullberg et al. (41) argued that there were no significant differences in cerebrospinal fluid biomarkers between patients who showed postoperative improvement and those who did not.

In addition to the above well-studied cerebrospinal fluid markers, researchers are looking for novel cerebrospinal fluid biomarkers that can diagnose and predict prognosis. Multiple studies have shown that neurofilament light chain (NFL) (42) is associated with axonal injury or degeneration. Furthermore, the concentration of NFL in the cerebrospinal fluid of iNPH patients is higher than that of healthy controls. However, Jeppsson et al. (43) reported opposite results, stating that there was no difference in NFL concentration between iNPH patients and healthy controls. There are also studies suggesting that glial fibrillary acidic protein (GFAP) could serve as a potential biomarker (44).

Some researchers have also investigated cerebrospinal fluid biomarkers for other neuronal injuries, including lipocalin-type prostaglandin D synthase (PGDS) (45), myelin basic protein (MBP) (46), protein tyrosine phosphatase receptor type Q (PTPRQ) (47), and various other molecules. However, these studies have produced contradictory results thus far.

Currently, there is substantial heterogeneity among studies investigating cerebrospinal fluid biomarkers. Various research cohorts have employed diverse inclusion and exclusion criteria, and their laboratory measurement techniques are inconsistently applied. Consequently, many studies have yielded inconsistent or conflicting findings. Further large-scale cohort studies are necessary to collectively establish a unified biomarker profile for normal pressure hydrocephalus in cerebrospinal fluid. This would enable clinicians to accurately diagnose the syndrome and tailor personalized treatment plans for each patient’s benefit.

Interestingly, the majority of researchers concentrate on biomarkers found in cerebrospinal fluid. However, we can leverage methodologies employed in the study of other neurodegenerative diseases, like Alzheimer’s disease, to investigate novel biomarkers. Xu et al. (48) explored immune cell infiltration and the expression patterns of genes related to immune function in Alzheimer’s disease patients. Employing machine learning algorithms, they identified five genes (PFKFB4, PDK3, KIAA0319L, CEBPD, and PHC2T) associated with immune cells and functions in AD, validating their accurate diagnostic potential. This implies potential for discovering or validating related genetic biomarkers for diagnosing NPH. In recent decades, the ongoing advancement of neuroimaging technologies, such as resting-state and task-based functional MRI, alongside electroencephalography, has facilitated the development of biomarkers for diagnosing cognitive and motor disorders.

Yin et al. (49) compared Alzheimer’s disease patients with normal controls and observed significant alterations in both the function and structure of the dorsal attention network among AD patients. Additionally, cognitive performance showed a close correlation with these observed alterations. Our comprehension of neurobrain network changes in NPH remains incomplete. Future research could utilize these advanced neuroimaging technologies to investigate potential neuroimaging biomarkers, which could offer substantial research insights.

### Limitations

This study is based on bibliometric and bibliographic visualization analyses of the literature, which can assist researchers in better understanding the developmental trends and academic frontiers of the field. However, this study has some limitations. Firstly, only English-language articles and reviews from SCI Expanded-indexed journals were included. Secondly, some details may be omitted due to the inability of VOSviewer and CiteSpace to analyze the full text of publications. Lastly, some newly published excellent articles may be excluded due to time lag.

## Conclusion

Bibliometric analysis revealed that current research on NHP is developing rapidly. The United States has contributed many outstanding scientific research achievements and breakthroughs in this field and ranked first among high output countries. Bibliometric analysis indicates that research on NHP is experiencing rapid growth. The USA has made significant contributions to scientific research and advancements in this area, ranking at the top among high output countries.

The university of Gothenburg has made novel progress and published most studies in this field. Better understanding the pathophysiological mechanisms of normal pressure hydrocephalus and identifying more accurate biomarkers in cerebrospinal fluid are hot topics for future research. The University of Gothenburg has achieved significant advancements and produced most researches in this area. Exploring the pathophysiological mechanisms of NPH and discovering more precise biomarkers in cerebrospinal fluid are important areas for future investigation.

## Author contributions

TC: Writing – original draft, Software, Visualization, Data curation, Formal analysis, Project administration. XH: Data curation, Software, Visualization, Writing – original draft. XZ: Software, Visualization, Writing – original draft. JL: Validation, Visualization, Writing – original draft. WB: Software, Visualization, Writing – original draft. JW: Conceptualization, Funding acquisition, Supervision, Writing – review & editing.
